# Fluid management of emergency department patients with sepsis—A survey of fluid resuscitation practices

**DOI:** 10.1111/aas.14141

**Published:** 2022-08-31

**Authors:** Marie Kristine Jessen, Birgitte Y. Simonsen, Marie‐Louise H. Thomsen, Lars W. Andersen, Jens Aage Kolsen‐Petersen, Hans Kirkegaard

**Affiliations:** ^1^ Research Center for Emergency Medicine, Department of Clinical Medicine Aarhus University and Aarhus University Hospital Aarhus Denmark; ^2^ Department of Emergency Medicine Aarhus University Hospital Aarhus Denmark; ^3^ Department of Anesthesiology and Intensive Care Aarhus University Hospital Aarhus Denmark; ^4^ Prehospital Emergency Medical Services Central Denmark Region Aarhus Denmark

**Keywords:** emergency department, fluid, questionnaire/survey, sepsis

## Abstract

**Background:**

Fluid administration and resuscitation of patients with sepsis admitted through emergency departments (ED) remains a challenge, and evidence is sparse especially in sepsis patients without shock. We aimed to investigate emergency medicine physicians' and nurses' perceptions, self‐reported decision‐making and daily behavior, and challenges in fluid administration of ED sepsis patients.

**Methods:**

We developed and conducted a multicenter, web‐based, cross‐sectional survey focusing on fluid administration to ED patients with sepsis sent to all nurses and physicians from the five EDs in the Central Denmark Region. The survey consisted of three sections: (1) baseline information; (2) perceptions of fluid administration and daily practice; and (3) clinical scenarios about fluid administration. The survey was performed from February to June, 2021.

**Results:**

In total, 138 of 246 physicians (56%) and 382 of 595 nurses (64%) responded to the survey. Of total, 94% of physicians and 97% of nurses regarded fluid as an important part of sepsis treatment. Of total, 80% of physicians and 61% of nurses faced challenges regarding fluid administration in the ED, and decisions were usually based on clinical judgment. The most common challenge was the lack of guidelines for fluid administration. Of total, 96% agreed that they would like to learn more about fluid administration, and 53% requested research in fluid administration of patients with sepsis. For a normotensive patient with sepsis, 46% of physicians and 44% of nurses administered 1000 ml fluid in the first hour. Of total, 95% of physicians and 89% of nurses preferred to administer ≥1000 ml within an hour if the patients' blood pressure was 95/60 at admission. There was marked variability in responses. Blood pressure was the most commonly used trigger for fluid administration. Respondents preferred to administer less fluid if the patient in the scenario had known renal impairment or heart failure. Normal saline was the preferred fluid.

**Conclusion:**

Fluid administration is regarded as an important but challenging aspect of sepsis management. Responses to scenarios revealed variability in fluid volumes. Blood pressure was the most used trigger. ED nurses and physicians request evidence‐based guidelines to improve fluid administration.

**Editorial Comment**

Fluid resuscitation of sepsis patients is complex, involving integration of both perceived patient needs and alternatives for fluid therapy. This study assessed practitioner choices through responses to hypothetical scenarios. Variation among responses from practitioners was observed, as would be expected.

## 1 BACKGROUND

Sepsis is one of the most common diagnoses in emergency department (ED) patients, and sepsis remains a major cause of morbidity and mortality.^1^, ^2^, ^3^ The mainstay of sepsis treatment includes intravenous antibiotics, source control, and fluids.^4^, ^5^ The physiology of fluid treatment, and the optimal volume of intravenous fluid, is not fully understood and elucidated.^6^ The Surviving Sepsis Campaign recommends giving at least 30 ml/kg to patients with sepsis‐induced hypotension or shock within the first 3 h. The recommendation is weak with low quality of evidence.^4^ No international recommendations for fluid management in patients with sepsis without hypotension and shock exist. Recent observational studies have found practice variation with the use of varying treatment regimens and fluid volumes, and evidence has been requested.^2^, ^7^, ^8^, ^9^

In the context of the low‐quality evidence to guide fluid administration and to better understand practice variation, we conducted a survey about fluid administration in patients with sepsis in the ED. The survey had two objectives: (1) to describe ED physicians' and nurses' perceptions of fluids, and how they regard their knowledge and confidence in handling fluid administration to patients with sepsis, and (2) to describe their daily decision‐making and practice regarding the quantity, infusion rate, and type of fluid administered in sepsis patients with varying comorbidities as well as the use of triggers to guide in fluid administration.

## 2 METHODS

### 2.1 Survey development

We conducted a multicenter, web‐based, cross‐sectional survey. The survey was developed according to the COSMIN guidelines.^10^ The research team consisting of researchers, physicians, and nurses with experience and expertise in ED and intensive care fluid administration. We generated items for the survey instrument through literature review.^11^, ^12^, ^13^, ^14^, ^15^, ^16^ Items were reduced and formatted to reduce respondent burden and increase the response rate. The survey was pilot tested by the research team and ED nurses and physicians, and then followed by a semi‐structured group interview. Participants in the group interview were five nurses and three physicians. The composition of the group and selection of participants was thoroughly chosen to reflect varying ages and experiences in both professions of the respondent group. The survey was assessed for clinical sensibility: whether the survey addressed how nurses and physicians administer fluids and whether it covered all aspects of the topic. The interview also focused on clinical clarity, face validity, and a target time to completion of maximum 15 min. The group interview was conducted using an interview‐guide and the interview was recorded and subsequently transcribed. Main topics of the interview were grouped and discussed. The survey was subsequently revised based on the provided feedback in collaboration with the group interview participants, who then tested the survey again.^17^

### 2.2 Survey content

The survey consisted of three sections: (1) baseline information regarding the participants; (2) perceptions of fluid administration and daily practice; and (3) scenarios about fluid administration, with a total of 32 questions. There were four hypothetical scenarios of a patient with sepsis and suspected pneumonia arriving in the ED: with normal blood pressure (BP: 120/75), with lower BP (BP: 95/60), with chronic renal failure, and with known heart failure. The survey instrument included five‐point Likert‐scale options for answers ranging from “strongly disagree” to “strongly agree” and questions with the possibility to choose multiple answers. Attitude‐based questions included a “Don't know” option. The survey was in Danish. A translated version is provided in Data S1.

### 2.3 Identification of study participants and survey distribution

We identified all ED physicians and registered nurses employed in the five EDs in the Central Denmark Region in February 2021 from local mailing lists. The five EDs provide 24‐h care to a mixed rural–urban population of 1.3 million people. In the five EDs, patient contacts vary between 15.000 and 63.000 per year.

The survey was structured and distributed through work emails using REDCap. Respondents activated the personal weblink and completed the survey instrument online. The survey was distributed from February to June 2021. To increase responses, non‐respondents received up to four email reminders.

Participation was voluntary, and consent for participation was implied by completion of the survey. The survey was anonymous. The study conduct was approved by the department managements of the participating departments. No ethical approval was required. The survey is reported in accordance with the Checklist of Reporting of Survey Studies (CROSS) (Data S1).^18^

### 2.4 Survey data collection and analysis

All data are presented as numbers and proportions for categorical variables. Missing responses were not imputed. The five‐point Likert scale responses were combined into three groups: “Agreed” (“Agreed” and “Strongly agreed”), “Neither/Or,” and “Disagreed” (“Disagreed” and “Strongly disagreed”) for purposes of data presentation in the manuscript but is shown in its full in tables. The data for all respondents were also described according to whether respondents were physicians or nurses. No sample size calculation was conducted a priori since the intent of the survey was to be descriptive and all physicians and nurses were contacted. We performed all analyses using Stata version 17 (StataCorp LP).

## 3 RESULTS

### 3.1 Study sample

The survey was distributed to 841 individuals employed in the EDs in Central Denmark Region: 246 physicians and 595 nurses. In total, 138 physicians (56%) and 382 nurses (64%) for a total of 520 (62%) responded to the survey. Characteristics of respondents are shown in Tables 1 and S1. In general, physicians had less ED working experience than nurses: 50% of physicians versus 16% of nurses had less than 1 year of ED working experience. Physicians had more recently been taught about fluid treatment in general: 32% of physicians and 7% of nurses had received formal education within the last year. For both groups, >30% never received formal education on fluid treatment or did not know.

**TABLE 1:** Characteristics of the respondents

	Physicians (*n* = 138)	Nurses (*n* = 382)
Age, *n* (%)		
20–30 years	53 (38%)	131 (34%)
31–40 years	42 (30%)	93 (24%)
41–50 years	25 (18%)	80 (21%)
51–60 years	14 (10%)	67 (28%)
61–70 years	4 (3%)	11 (3%)
Sex, *n* (%)		
Male	57 (41%)	17 (4%)
Female	79 (57%)	364 (95%)
Other	2 (1%)	1 (0%)
Emergency department working experience, *n* (%)		
0–11 months	69 (50%)	61 (16%)
1–2 years	19 (14%)	67 (18%)
3–4 years	17 (12%)	64 (17%)
5–7 years	15 (11%)	61 (16%)
8+ years	18 (13%)	129 (34%)
Most recent educational session on fluid treatment, *n* (%)		
0–6 months	27 (22%)	9 (3%)
7–12 months	12 (10%)	13 (4%)
>12 months	44 (36%)	127 (40%)
Never	32 (26%)	119 (38%)
Do not know	8 (7%)	49 (15%)
Physician status, *n* (%)		
House officer (<1 year of experience)	41 (30%)	‐
Senior House Officer (>1 year of experience)	23 (17%)	
Specialist Registrar (>2 years of experience)	29 (21%)	
Senior Registrar (>5 years of experience)	13 (9%)	
Consultant	30 (22%)	
Other	2 (1%)	

### 3.2 Perceptions of fluid administration

87% of physicians and 82% of nurses agreed that they regarded fluid as medication with potential side effects. Of total, 94% of physicians and 97% of nurses indicated that fluid is an important part of the treatment of sepsis. The majority agreed that they felt confident in managing fluid treatment of patients with sepsis (physicians: 70%, nurses: 83%) while fewer felt confident in managing fluid treatment of patients in septic shock (46% and 59%, respectively) (Table 2). Of total, 12% of physicians and 6% of nurses did not feel confident in managing fluid administration to patients with sepsis and 36% of physicians and 20% of nurses did not feel confident in managing fluid administrations to patients with septic shock.

**TABLE 2:** Survey responses: perceptions of fluid administration

	Physicians (*n* = 138)	Nurses (*n* = 382)
“I regard intravenous fluid as medication,” *n* (%)		
Strongly agree	56 (41%)	121 (32%)
Agree	63 (46%)	185 (50%)
Neither or	14 (10%)	55 (15%)
Disagree	4 (3%)	12 (3%)
Strongly disagree	1 (1%)	2 (0%)
“Intravenous fluids can have side effects,” *n* (%)		
Strongly agree	87 (63%)	185 (49%)
Agree	45 (33%)	169 (45%)
Neither or	3 (2%)	15 (4%)
Disagree	2 (1%)	6 (2%)
Strongly disagree	0	0
“Besides intravenous antibiotics, the treatment of sepsis is fluid,” *n* (%)		
Strongly agree	70 (51%)	210 (56%)
Agree	60 (43%)	152 (41%)
Neither or	7 (5%)	12 (3%)
Disagree	1 (1%)	1 (0%)
Strongly disagree	0	0
“I am confident and have the skills to manage fluid treatment of patients with sepsis,” *n* (%)		
Strongly agree	30 (22%)	106 (29%)
Agree	66 (48%)	199 (54%)
Neither or	25 (18%)	45 (12%)
Disagree	15 (11%)	22 (6%)
Strongly disagree	1 (1%)	0
“I am confident and have the skills to manage fluid treatment of patients with septic shock,” *n* (%)		
Strongly agree	18 (13%)	55 (15%)
Agree	45 (33%)	164 (44%)
Neither or	25 (18%)	74 (20%)
Disagree	40 (29%)	68 (18%)
Strongly disagree	9 (7%)	10 (3%)

#### 3.2.1 Administration of fluids and use of guidelines

There did not seem to be any consensus on how and by whom intravenous fluids were prescribed (Table 3). Of total, 63% of physicians and 58% of nurses reported that fluids were administered without a formal physician prescription either daily or frequently within a given week. Some of the EDs had local regulations allowing nurses to administer a restricted amount of fluid without a prescription by a physician. Of total, 86% reported that prescriptions “never,” “rarely,” or “sometimes” included infusion rates (in ml/h) (physicians: 79%, nurses: 89%) (Table 3).

**TABLE 3:** Survey responses: administration of fluids and use of guidelines

	Physicians (*n* = 138)	Nurses (*n* = 382)
Prescription and administration of fluids in the ED		
All fluids are prescribed by physicians before administration	23 (17%)	38 (10%)
On a weekly basis, fluids are administered without physician	42 (31%)	66 (18%)
prescription	43 (32%)	145 (40%)
Daily, fluids are administered without physician prescription	19 (14%)	114 (31%)
Fluids are administered by nurses according to local guidelines	9 (7%)	3 (1%)
Do not know		
Infusion rates—“How often do prescriptions of fluid describe the infusion rate (ml/h)?”		
Always	2 (1%)	0
Often	21 (15%)	37 (10%)
Sometimes	72 (53%)	190 (52%)
Rarely	34 (25%)	127 (35%)
Never	2 (1%)	7 (2%)
Do not know	5 (4%)	5 (1%)
Experienced challenges in fluid administration in the ED^a^		
No challenges	11 (8%)	92 (25%)
No time to consider fluid treatment for each patient	40 (29%)	57 (15%)
No time to administer the fluids (ensure iv. access, hang fluids, documentation etc.)	13 (9%)	43 (11%)
No guidelines for fluid administration	74 (54%)	125 (33%)
Lack of evidence within the field of fluids in sepsis	44 (32%)	43 (11%)
Large heterogeneity in sepsis patients (e.g., different infectious foci or comorbidity)	42 (30%)	53 (14%)
Other challenges^b^	4 (3%)	14 (4%)
Do not know	17 (12%)	51 (14%)
“Does a helpful guideline in fluid administration to sepsis patients in your ED exist,” *n* (%)		
Yes, and I use it	62 (46%)	142 (39%)
Yes, but I do not use it/it does not help me	25 (18%)	77 (21%)
No, there is no guideline	9 (7%)	20 (6%)
Do not know	40 (29%)	124 (34%)
“Would you prefer to have a helpful guideline in fluid administration of patients with sepsis,” *n* (%)^c^		
Yes	62 (84%)	161 (73%)
No	4 (5%)	9 (4%)
Do not know	8 (11%)	51 (23%)

*Note*: All data are presented as *n* (%).

Abbreviation: ED, emergency department.

^a^ It was possible to check more than one option if “No challenges” was not checked.

^b^ Reasons most often describing challenges about documentation of fluids and nurses describing, that physicians do not decide on fluids for all patients.

^c^ The question was only available, if the above question was answered “Yes, but I don't use it/it doesn't help me,” “No, there is no guideline,” or “Do not know.”

Overall, 80% of physicians and 61% of nurses faced challenges in fluid administration. For both groups, the most common challenge was the lack of guidelines for fluid administration, and for physicians, especially, also lack of evidence within the field, large heterogeneity in patients with sepsis, and no time to consider fluid treatment for each patient. Of total, 54% of physicians and 61% of nurses reported, that there was either no local guideline, the existing guidelines did not help them in their clinical work, or they did not know if there was a guideline. Of these, the majority of both physicians and nurses would prefer to have a guideline, that could assist them in their clinical work (Table 3). Also, 99% of physicians and 95% of nurses agreed that they preferred to learn more about fluid administration, and 68% and 48%, respectively, requested research within the field of fluid administration and treatment of patients with sepsis (Table S2).

### 3.3 Use of triggers in fluid administration

**FIGURE 1:**
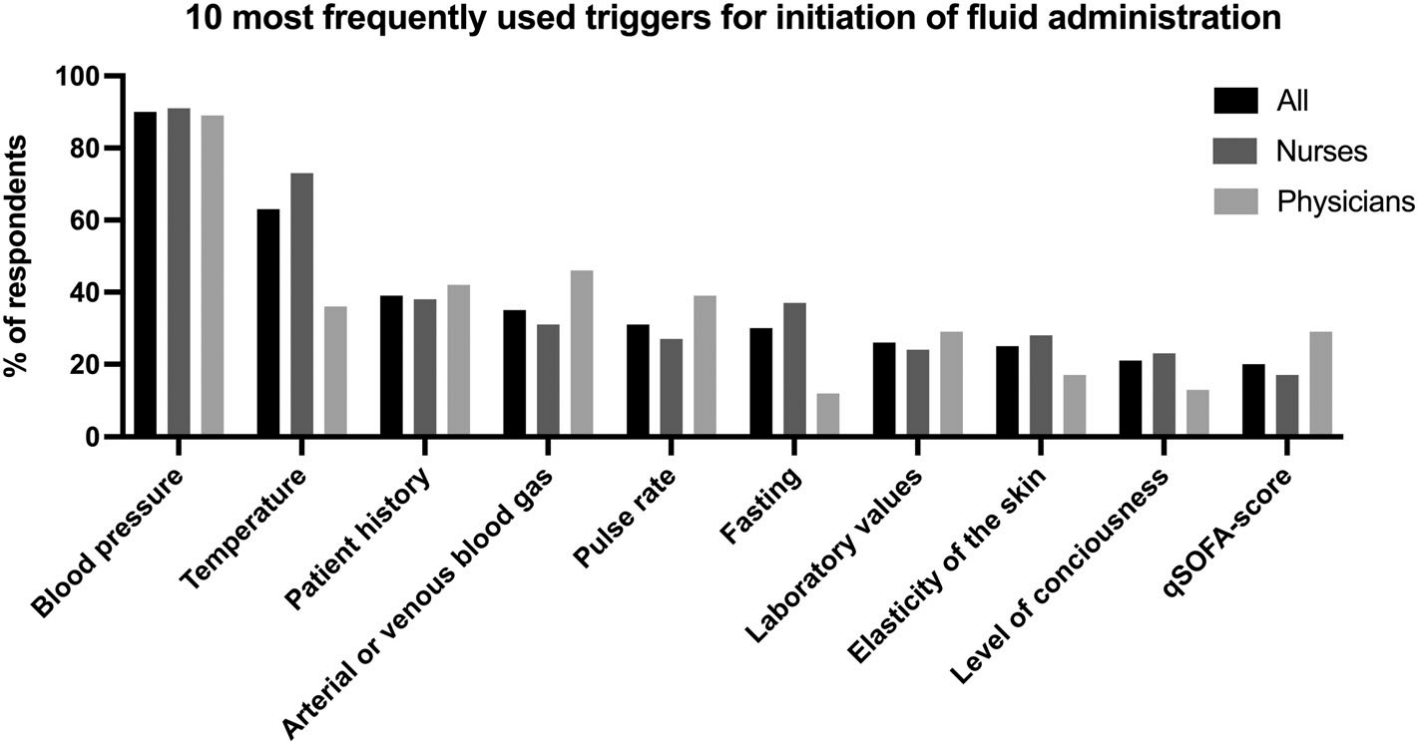
The 10 most frequently used triggers for initiation of fluids within 60 min of patient admission by respondent groups reported in percentage. Laboratory values included creatinine, albumin, and so forth. qSOFA, quick SOFA‐score (sequential organ failure assessment score)

**FIGURE 2:**
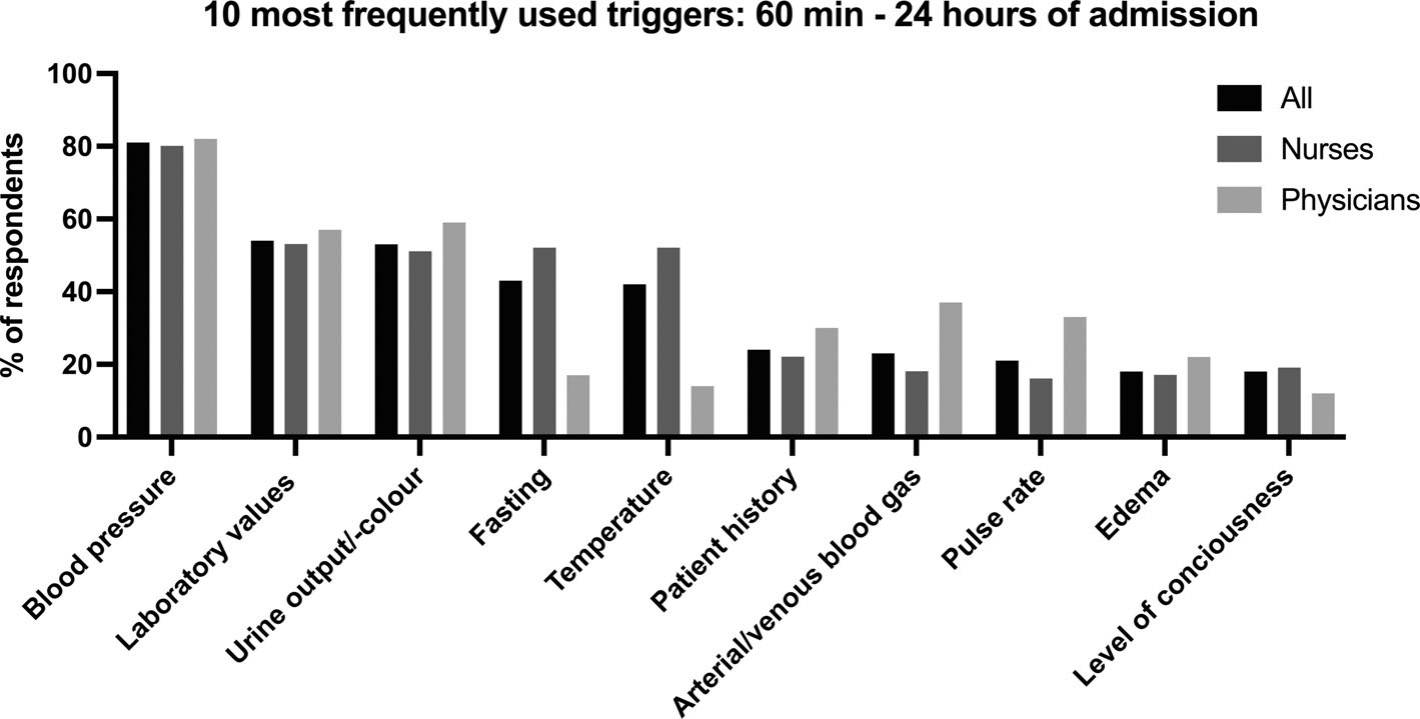
The 10 most frequently used triggers to evaluate fluid response and further administration from 60 min to 24 h of patient admission by respondent groups reported in percentage. Laboratory values included creatinine, albumin, and so forth.

The most frequently used triggers to initiate fluids at patient arrival were blood pressure (90%), temperature (63%), patient history (39%), and arterial/venous blood gas analyses including lactate measurements (35%) (Figures 1 and S1; Table S4). The most frequently used triggers to evaluate fluid response and further administration from 60 min until 24 h of patient arrival were blood pressure (81%), laboratory values (54%), urine output and color (53%), and fasting (43%) (Figures 2 and S1; Table S4). Data on use of triggers in physicians and nurses separately are provided in Table S4 and Figure S1.

### 3.4 Clinical scenarios

In a clinical scenario describing an otherwise healthy 55‐year‐old woman (70 kg) with sepsis due to suspected pneumonia, the most common choice (physicians: 46% and nurses: 44%) was to administer 1000 ml intravenous fluids within 1 h. Of total, 37% of physicians and 38% of nurses would prefer to administer 500 ml in 1 h. When the same patient was admitted with lower blood pressure (95/60 mmHg), the preferred fluid volumes in general increased, and 95% of physicians and 89% of nurses preferred to administer ≥1000 ml within an hour of admission to this patient (Table 4). Of total, 86% of physicians and 79% of nurses answered that the latter patient should have the fluid status reevaluated within a maximum of 1 h (Table 4).

**TABLE 4:** Clinical scenarios

	Physicians (*n* = 138)	Nurses (*n* = 382)
Case 1: A previously, otherwise healthy 55‐year‐old woman (70 kg) was admitted with suspected pneumonia, with a history of cough and dyspnea through 14 days. She is slightly confused (GCS 14), BP 120/75, pulse rate 120, respiration rate 28, temperature 39.1°C and saturation 92% (3 L/min nasal oxygen). How much fluid would you administer within the first hour?		
No fluids	10 (8%)	38 (11%)
500 ml	46 (37%)	127 (38%)
1000 ml	58 (46%)	147 (44%)
1500 ml	4 (3%)	6 (2%)
2000	4 (3%)	9 (3%)
3000	2 (2%)	0
4000	0	0
Do not know	1 (1%)	8 (2%)
Case 2: As in Case 1, but BP 95/60. How much fluid would you administer within the first hour?		
No fluids	0	1 (0%)
500 ml	6 (5%)	28 (9%)
1000 ml	65 (53%)	205 (63%)
1500 ml	15 (12%)	33 (10%)
2000	33 (27%)	47 (14%)
3000	4 (3%)	6 (2%)
4000	0	1 (0%)
Do not know	0	5 (2%)
Case 3: As in Case 1, but with known renal failure (habitual creatinine 200–220 μmol/L). How much fluid would you administer within the first hour?		
No fluids	12 (10%)	60 (19%)
500 ml	45 (37%)	109 (34%)
1000 ml	40 (40%)	71 (22%)
1500 ml	3 (2%)	7 (2%)
2000	5 (4%)	7 (2%)
3000	0	3 (1%)
4000	1 (1%)	0
Do not know	8 (7%)	65 (20%)
Case 4: As in Case 1, but with known heart failure (ejection fraction usually 30%). How much fluid would you administer within the first hour?		
No fluids	36 (29%)	152 (48%)
500 ml	58 (47%)	109 (34%)
1000 ml	16 (13%)	20 (6%)
1500 ml	1 (1%)	0
2000	2 (2%)	1 (0%)
3000	0	1 (0%)
4000	1 (1%)	0
Do not know	9 (7%)	35 (11%)
Case 1 and 2: Preferred fluids if electrolytes are normal		
NaCl (0.9% normal saline)	51 (42%)	156 (48%)
Ringers' (acetate or lactate)	27 (22%)	40 (12%)
Initially NaCl followed by Ringers'	43 (35%)	108 (33%)
NaCl and albumin	0	0
Ringers' and albumin	0	2 (1%)
Kalium‐natrium‐glucose	1 (1%)	2 (1%)
Glucose	1 (1%)	0
Do not know	0	17 (5%)
Cases 1 and 2: What did you base your decisions on fluid volumes on?		
Knowledge and evidence in the field	16 (13%)	46 (14%)
Clinical judgment	65 (52%)	165 (52)
Education	6 (5%)	21 (6%)
Experience	23 (18%)	64 (19%)
Patient history	11 (8%)	31 (9%)
Other	6 (4%)	8 (2%)
Case 2: The patient's fluid status should be reevaluated after		
1 h	106 (86%)	257 (79%)
2–3 h	16 (13%)	59 (18%)
4–5 h	0	8 (2%)
6–12 h	0	1 (0%)
13–24 h	1 (1%)	1 (0%)

*Note*: All data are presented as *n* (%).

Abbreviation: BP, blood pressure.

For a similar patient with normal blood pressure but with known renal impairment (habitual creatinine 200–220 μmol/L), overall fluid administration decreased. The respondent proportion preferring to give no fluids increased (physicians: 10%, nurses: 10%), as well as the respondent proportion who did not know (physicians: 7%, nurses: 20%) (Table 4). This was also seen in the scenario with a patient with known heart failure with usual ejection fraction of 30%. To this patient, physicians preferred giving 500 ml in 1 h (physicians: 47%, nurses: 34%), whereas nurses preferred not giving fluids at all (physicians: 29%, nurses: 48%).

Most respondents would administer normal saline to the patients (physicians: 42%, nurses: 48%) or “Initially NaCl followed by Ringers” (physicians: 35%, nurses: 33%). The decisions on fluid administration in the scenarios were primarily based on a clinical judgment (i.e., intuition, previous knowledge, and experiences combined with bedside examinations and/or observations) (Table 4).

## 4 DISCUSSION

The survey demonstrated that fluid is regarded an important part of sepsis treatment in Danish EDs. Most nurses and physicians faced challenges when managing fluid administration, such as deciding on the volume, appropriate triggers, rates of intravenous fluids. A significant proportion did not feel confident in managing fluids to patients with sepsis or septic shock. Lack of evidence‐based guidelines leaves the treating teams with difficult decisions based on primarily clinical judgment, resulting in considerable variability in choices on volumes of fluid administered in the clinical scenarios.

Fluid administration remains, despite its frequent in‐hospital use, a controversial topic in critical care and emergency medicine leading to uncertainty regarding best practice.^6^ There is no firm evidence, no measurement or laboratory value, and so forth, to guide in fluid treatment with certainty. This uncertainty is reflected in the results of the survey by marked variation in attitudes toward the volume of fluid administration in the clinical scenarios and more than 20% of respondents not feeling confident in managing fluid administration to patients with sepsis. Another study, likewise, found that respondents had to rely on previous knowledge and experiences combined with bedside observations.^19^

We found a lower proportion of respondents feeling confident in administering fluid to septic shock than to patients with sepsis (physicians: 70% and nurses: 83% vs. physicians: 46% and nurses: 59%), which may reflect insecurity toward the sickest patients. However, the finding is in contrast to a similar survey from Denmark investigating fluid management in the prehospital setting.^20^ Also, it appears contradictory to the fact that recommendations for fluid administration in septic shock/sepsis‐associated hypotension do exist (Surviving Sepsis Campaign: 30 ml/kg in 3 h), although a “weak recommendation (with) low quality of evidence”^4^ whereas there are no recommendations in sepsis without hypotension. Overall, less respondents felt confident in administering fluid to both sepsis and septic shock, compared to prehospital critical care anesthesiologist (100%, 100%) and ambulance personnel (73%, 79%).^20^ Furthermore, physicians felt less confident than nurses. This may reflect limited ED working experience for the physician respondents or a higher awareness of the lacking evidence‐based recommendations/guidelines among physicians compared to nurses. A previous scoping review described that especially junior physicians regard prescribing fluid as an “inherently complex ‘whole task’” and with lack of prescribing education.^21^ In an interview study, participants in general struggled to articulate their decision‐making process regarding specific timing and volume of fluid resuscitation, which is in line with the findings of the current study.^19^ Both nurses and physicians requested (improved) evidence‐based guidelines.

Blood pressure was the most frequently used trigger for fluid initiation and further fluid administration, in accordance with self‐perceived practice in the prehospital setting.^20^ Several ICU‐based studies have found hypotension/blood pressure to be the primary indication for giving fluid boluses.^22^, ^23^, ^24^ Interestingly, the use of triggers changed from initial fluid assessment and initiation (within the first 60 min) to further fluid administration (60 min to 24 h). For the more long‐term fluid assessment, laboratory values (creatinine, hemoglobin, albumin, etc.), urine output, and fasting were considered essential. In the initial phase, temperature, patient history (e.g., fluid loss prior to admission), and lactate measurements were considered more important to guide fluid therapy.

Respondents were prone to administer less fluid to a hypothetical patient with known renal impairment compared to one without comorbidity. We did not further investigate the reasons for this decision. In a survey investigating de‐resuscitation practice, they found respondents to be less likely to administer diuretics to a patient in acute renal failure.^13^ We may speculate that both may reflect uncertainty in treatment of patients with known or acute renal failure or impairment, due to sparse evidence.

For patients with known heart failure, respondents also administered less fluids. Accordingly, a survey found that respondents would administer less fluid in patients in septic shock with heart failure with reduced ejection fraction <40%, compared to those without heart failure, before deeming the patient volume non‐responsive.^25^ None of the included EDs were a part of the, at that time, ongoing CLASSIC‐trial investigating restrictive fluids in ICU patients with septic shock^26^; however, we acknowledge that the general perception of fluid volumes may be changing toward a more restrictive approach, which may have affected responses to questions of fluid volumes in the clinical scenarios. Of note, 31% of physicians responded that use of early vasopressors administered through a peripheral intravenous catheter in the ED could be implemented as an alternative to fluids (Table S3).

Normal saline was the preferred fluid of choice. This is in accordance with the findings in other surveys in emergency medicine,^15^, ^27^ although practice seems to be changing toward using more balanced fluids.^28^ For the sites in the survey having local regulations allowing nurses to administer a restricted amount of fluid, these only allowed for administration of normal saline, possibly affecting their response toward choosing normal saline over other fluids.

Ongoing studies investigating fluid administration strategies in patients with sepsis‐associated hypotension and septic shock in EDs and ICUs are awaited.^29^, ^30^, ^31^ However, there is a paucity of studies investigating fluid treatment of ED sepsis patients without hypotension, accounting for the largest proportion of ED patients. This survey—to the best of our knowledge, is the first to survey fluids in sepsis patients without shock—emphasized that ED physicians and nurses are eager to learn more about the field, and that research within the field of fluid administration to patients not in shock is requested.

### 4.1 Limitations

This survey has not been validated as a measurement instrument; however, the survey was conducted in accordance with guidelines from COSMIN and the purpose was to reflect the daily clinical practice of handling fluids in Danish EDs. The generalizability to other countries in general is uncertain. Emergency medicine is a new specialty in Denmark. As such, administering for example vasopressors for now still requires assistance from anesthesiologists and usually transfer to an intensive care unit. This may have caused a tendency to administer more fluids instead of considering early vasopressors. The thoughts on especially triggers are probably universal. The main limitation of this survey is the low response rate, which raises concerns that the sample does not represent the entire ED staff population. The survey was distributed during the COVID‐19 pandemic, which could have affected the response rate. It is possible that respondents are those with more interest in the topic and potentially more polarized views. The questions on fluid administration were asked using hypothetical clinical scenarios in early sepsis, reflecting self‐reported clinician behaviors, which may not reflect true practice management. Also, the scenarios only described the early treatment of sepsis not covering later stages of sepsis during hospital admission.

In conclusion, fluid administration is regarded an important but difficult aspect of sepsis treatment. Nurses and physicians primarily base their decisions on clinical judgment, and they request evidence‐based guidelines to improve fluid management. Responses to scenarios revealed variability in fluid volumes, with no consensus on appropriate indications, volumes, or rates, although blood pressure was the most frequently used trigger. These findings support further investigations within the field to provide specific recommendations in guidelines.

## AUTHOR CONTRIBUTIONS

Marie Kristine Jessen, Birgitte Y. Simonsen, and Marie‐Louise H. Thomsen developed and tested the survey. Lars W. Andersen, Jens A. K. Petersen, and Hans Kirkegaard tested the survey and provided expert knowledge in the conduction process. Marie Kristine Jessen drafted the manuscript. All authors approved the submission of the manuscript.

## CONFLICT OF INTEREST

The authors declare no conflict of interest.

## Supporting information


**Data S1** Supporting Information

## References

[1] Henriksen DP , Laursen CB , Jensen TG , Hallas J , Pedersen C , Lassen AT . Incidence rate of community‐acquired sepsis among hospitalized acute medical patients‐a population‐based survey. Crit Care Med. 2015;43(1):13‐21. 25251760 10.1097/CCM.0000000000000611

[2] Jessen MK , Andersen LW , Thomsen MH , et al. Twenty‐four‐hour fluid administration in emergency department patients with suspected infection: a multicenter, prospective, observational study. Acta Anaesthesiol Scand. 2021;65(8):1122‐1142. 33964019 10.1111/aas.13848

[3] Rudd KE , Johnson SC , Agesa KM , et al. Global, regional, and national sepsis incidence and mortality, 1990‐2017: analysis for the Global Burden of Disease Study. Lancet. 2020;395(10219):200‐211. 31954465 10.1016/S0140-6736(19)32989-7 PMC6970225

[4] Evans L , Rhodes A , Alhazzani W , et al. Surviving sepsis campaign: international guidelines for management of sepsis and septic shock 2021. Crit Care Med. 2021;49(11):e1063‐e1143. 34605781 10.1097/CCM.0000000000005337

[5] Levy MM , Evans LE , Rhodes A . The surviving sepsis campaign bundle: 2018 update. Intensive Care Med. 2018;44(6):925‐928. 29675566 10.1007/s00134-018-5085-0

[6] Myburgh JA , Mythen MG . Resuscitation fluids. N Engl J Med. 2013;369(13):1243‐1251. 24066745 10.1056/NEJMra1208627

[7] Marik PE , Linde‐Zwirble WT , Bittner EA , Sahatjian J , Hansell D . Fluid administration in severe sepsis and septic shock, patterns and outcomes: an analysis of a large national database. Intensive Care Med. 2017;43(5):625‐632. 28130687 10.1007/s00134-016-4675-y

[8] Harris T , Coats TJ , Elwan MH . Fluid therapy in the emergency department: an expert practice review. Emerg Med J. 2018;35(8):511‐515. 29807929 10.1136/emermed-2017-207245

[9] Keijzers G , Macdonald SP , Udy AA , et al. The Australasian resuscitation in sepsis evaluation: fluids or vasopressors in emergency department sepsis (ARISE FLUIDS), a multi‐centre observational study describing current practice in Australia and New Zealand. Emerg Med Australas. 2020;32(4):586‐598. 32043315 10.1111/1742-6723.13469 PMC7496107

[10] Mokkink LB , Terwee CB , Knol DL , et al. The COSMIN checklist for evaluating the methodological quality of studies on measurement properties: a clarification of its content. BMC Med Res Methodol. 2010;10:22. 20298572 10.1186/1471-2288-10-22 PMC2848183

[11] van den Hengel LC , Visseren T , Meima‐Cramer PE , Rood PP , Schuit SC . Knowledge about systemic inflammatory response syndrome and sepsis: a survey among Dutch emergency department nurses. Int J Emerg Med. 2016;9(1):19. 27416936 10.1186/s12245-016-0119-2 PMC4945519

[12] Rahman NA , Chan CM , Zakaria MI , Jaafar MJ . Knowledge and attitude towards identification of systemic inflammatory response syndrome (SIRS) and sepsis among emergency personnel in tertiary teaching hospital. Australas Emerg Care. 2019;22(1):13‐21. 30998867 10.1016/j.auec.2018.11.002

[13] Silversides JA , McAuley DF , Blackwood B , Fan E , Ferguson AJ , Marshall JC . Fluid management and deresuscitation practices: a survey of critical care physicians. J Intensive Care Soc. 2020;21(2):111‐118. 32489406 10.1177/1751143719846442 PMC7238475

[14] Storozuk SA , MacLeod MLP , Freeman S , Banner D . A survey of sepsis knowledge among Canadian emergency department registered nurses. Australas Emerg Care. 2019;22(2):119‐125. 31042531 10.1016/j.auec.2019.01.007

[15] McIntyre L , Rowe BH , Walsh TS , et al. Multicountry survey of emergency and critical care medicine physicians' fluid resuscitation practices for adult patients with early septic shock. BMJ Open. 2016;6(7):e010041. 10.1136/bmjopen-2015-010041 PMC4947761 27388345

[16] McIntyre LA , Hébert PC , Fergusson D , Cook DJ , Aziz A . A survey of Canadian intensivists' resuscitation practices in early septic shock. Crit Care. 2007;11(4):R74. 17623059 10.1186/cc5962 PMC2206518

[17] Mokkink LB , Terwee CB , Patrick DL , et al. The COSMIN checklist for assessing the methodological quality of studies on measurement properties of health status measurement instruments: an international Delphi study. Qual Life Res. 2010;19(4):539‐549. 20169472 10.1007/s11136-010-9606-8 PMC2852520

[18] Sharma A , Minh Duc NT , Luu Lam Thang T , et al. A consensus‐based checklist for reporting of survey studies (CROSS). J Gen Intern Med. 2021;36(10):3179‐3187. 33886027 10.1007/s11606-021-06737-1 PMC8481359

[19] Fluid resuscitation in septic shock: an exploration of emergency department and critical care clinician perceptions and decision‐making. 2020. Accessed 28 August, 2022. https://410medical.com/wp‐content/uploads/2020/09/Fluid‐Resuscitation‐in‐Septic‐Shock‐Perceptions‐White‐Paper‐Sept‐2020‐1.pdf.

[20] Jensen ME , Jensen AS , Meilandt C , et al. Prehospital fluid therapy in patients with suspected infection: a survey of ambulance personnel's practice. Scand J Trauma Resusc Emerg Med. 2022;30(1):38. 35642066 10.1186/s13049-022-01025-1 PMC9158174

[21] McCrory RFR , Gormley GJ , Maxwell AP , Dornan T . Learning to prescribe intravenous fluids: a scoping review. Perspect Med Educ. 2017;6(6):369‐379. 29119469 10.1007/s40037-017-0386-5 PMC5732109

[22] Cecconi M , Hofer C , Teboul JL , et al. Fluid challenges in intensive care: the FENICE study: a global inception cohort study. Intensive Care Med. 2015;41(9):1529‐1537. 26162676 10.1007/s00134-015-3850-x PMC4550653

[23] Bjerregaard MR , Hjortrup PB , Perner A . Indications for fluid resuscitation in patients with septic shock: post‐hoc analyses of the CLASSIC trial. Acta Anaesthesiol Scand. 2019;63(3):337‐343. 30318584 10.1111/aas.13269

[24] Bihari S , Prakash S , Bersten AD . Post resusicitation fluid boluses in severe sepsis or septic shock: prevalence and efficacy (price study). Shock. 2013;40(1):28‐34. 23635850 10.1097/SHK.0b013e31829727f1

[25] Wardi G , Joel I , Villar J , et al. Equipoise in appropriate initial volume resuscitation for patients in septic shock with heart failure: results of a multicenter clinician survey. J Intensive Care Med. 2020;35(11):1338‐1345. 31446829 10.1177/0885066619871247 PMC7039763

[26] Meyhoff TS , Hjortrup PB , Wetterslev J , et al. Restriction of intravenous fluid in ICU patients with septic shock. N Engl J Med. 2022;386:2459‐2470. 35709019 10.1056/NEJMoa2202707

[27] Jiwaji Z , Brady S , McIntyre LA , Gray A , Walsh TS . Emergency department management of early sepsis: a national survey of emergency medicine and intensive care consultants. Emerg Med J. 2014;31(12):1000‐1005. 24005642 10.1136/emermed-2013-202883

[28] Jacobs K , Hindle L , Omar S . A review of adult resuscitative fluid purchasing and usage trends at an academic tertiary hospital in Johannesburg. Anaesthesiol Intensive Ther. 2021;54:56‐61. 10.5114/ait.2021.111343 PMC10156498 34870384

[29] Meyhoff TS , Hjortrup PB , Møller MH , et al. Conservative vs liberal fluid therapy in septic shock (CLASSIC) trial‐protocol and statistical analysis plan. Acta Anaesthesiol Scand. 2019;63(9):1262‐1271. 31276193 10.1111/aas.13434

[30] Self WH , Semler MW , Bellomo R , et al. Liberal versus restrictive intravenous fluid therapy for early septic shock: rationale for a randomized trial. Ann Emerg Med. 2018;72:457‐466. 29753517 10.1016/j.annemergmed.2018.03.039 PMC6380679

[31] Macdonald SPJ , Keijzers G , Taylor DM , et al. Restricted fluid resuscitation in suspected sepsis associated hypotension (REFRESH): a pilot randomised controlled trial. Intensive Care Med. 2018;44(12):2070‐2078. 30382308 10.1007/s00134-018-5433-0

