# Supplementary material for: Fluid management of emergency department patients with sepsis—A survey of fluid resuscitation practices
Source: Acta Anaesthesiol Scand. 2022 Aug 31;66(10):1237–46. doi: 10.1111/aas.14141 (PMC9805143; doi:10.1111/aas.14141)
Supplement: Supplementary file 1 — Data S1 Supporting Information [file AAS-66-1237-s001.pdf]

SUPPLEMENTARY CONTENT S1

Fluid management and resuscitation for adult patients with suspected infection and sepsis – a survey of emergency department physicians' and nurses' fluid resuscitation practices

Survey

Translated version of the original survey – original version was in Danish

Title: Fluid administration in patients with suspected infection and sepsis

**Introduction**		
	How old are you?	○ 20-30 years<br>○ 31-40 years<br>○ 41-50 years<br>○ 51-60 years<br>○ 61-70 years<br>○ >70 years
	What is your gender?	○ Male<br>○ Female<br>○ Other
	My education is as	○ Nurse<br>○ Medical doctor<br>○ Other
	For medical doctors only:<br>Physician status:	○ Foundation year<br>○ Internship<br>○ Residency<br>○ Junior attending<br>○ Senior attending<br>○ Other
	My primary specialty (full training completed or currently in training in the following…)	○ All 39 specialties acknowledged in Denmark were listed
	For nurses only:<br>Do you have further training in nursing (besides nursing school)?	○ No<br>○ Yes, foundation module of emergency medicine nursing specialization<br>○ Yes, foundation and module 2 of emergency medicine nursing specialization<br>○ Yes, alle three modules of emergency medicine nursing specialization<br>○ Yes, specialized in aneasthesia<br>○ Ja, specialized in critical care

		○ Other
	For all respondents:<br>How many years of emergency department experience do you have?	○ 0-11 months<br>○ 1-2 years<br>○ 3-4 years<br>○ 5-7 years<br>○ 8+ years
	I work in the ED at the following hospital:	○ Regional Hospital Randers<br>○ Regional Hospital Viborg<br>○ Regional Hospital Herning<br>○ Regional Hospital Horsens<br>○ Aarhus University Hospital
**Perceptions of fluid administration – statements and daily practice in your department**<br><br>The following questions are about your perceptions of and the daily use of fluids in your department. Intravenous fluids are regarded as "clear fluids" i.e., crystalloids/colloids ex. Normal saline, Ringer etc. but not blood products.		
	I regard intravenous fluid as medication	○ Strongly agree<br>○ Agree<br>○ Neither or<br>○ Disagree<br>○ Strongly disagree
	Intravenous fluids can have side effects	○ Strongly agree<br>○ Agree<br>○ Neither or<br>○ Disagree<br>○ Strongly disagree
	The treatment of sepsis is fluids, besides intravenous antibiotics	○ Strongly agree<br>○ Agree<br>○ Neither or<br>○ Disagree<br>○ Strongly disagree
	I am confident and have the skills to manage fluid treatment of patients with sepsis*	○ Strongly agree<br>○ Agree<br>○ Neither or

	* Definition of sepsis: suspected infection + organ dysfunction	○ Disagree<br>○ Strongly disagree
	I am confident and have the skills to manage fluid treatment of patients with septic shock**<br><br>** Definition of septic shock: Suspected infection + lactate ≥ 2 mmol/l + need for vasopressors to maintain blood pressure despite fluid administration	○ Strongly agree<br>○ Agree<br>○ Neither or<br>○ Disagree<br>○ Strongly disagree
	Prescription and administration of fluids in the ED	○ All fluids are prescribed by physicians before administration<br>○ Weekly fluids are administered without physician prescription<br>○ Daily fluids are administered without physician prescription<br>○ Fluids are administered by nurses according to local guidelines<br>○ Don't know
	Infusion rates – "How often do prescriptions of fluid describe the infusion rate (ml/h)?"	○ Always<br>○ Often<br>○ Sometimes<br>○ Rarely<br>○ Never<br>○ Don't know
	Experienced challenges in fluid administration in the ED<br>(Possible to check more than one option)	○ No challenges<br>○ No time to consider fluid treatment for each patient<br>○ No time to administer the fluids (ensure iv. access, hang fluids, documentation etc.)<br>○ No guidelines for fluid administration<br>○ Lack of evidence within the field of fluids in sepsis<br>○ Large heterogeneity in sepsis patients (i.e. different infectious foci or comorbidity)

		○ Other challenges<br>○ Don’t know
	Does a helpful guideline in fluid administration to sepsis patients in your ED exist	○ Yes, and I use it<br>○ Yes, but I don’t use it/it doesn’t help me<br>○ No, there is no guideline<br>○ Don’t know
	Would you prefer to have a helpful guideline in fluid administration of patients with infections	○ Yes<br>○ No<br>○ Don’t know
	Choose the 5 triggers, you most often use to initiate fluids during the first 60 min of admission	○ Pulse rate<br>○ Arterial or venous blood gas including lactate<br>○ Fasting<br>○ Capillary refill time<br>○ Elasticity of the skin<br>○ Blood pressure<br>○ Mean arterial pressure<br>○ SOFA-score<br>○ Passive leg raise<br>○ Jugular vein distension<br>○ Edema<br>○ Stethoscopy pulmonis<br>○ Laboratory values i.e., creatinine, albumin etc.<br>○ Mottling<br>○ Level of consciousness<br>○ Mucus membranes<br>○ Ultrasound of heart, lungs, or vena cava inferior<br>○ Patient history<br>○ Temperature of the extremities<br>○ qSOFA-score<br>○ Urine output or colour<br>○ Respiratory rate<br>○ Temperature (oral, rectal or in-ear)

	Do you use other triggers for fluid administration during the first 60 min of admission?	Add others (text)
	Choose the 5 triggers, you most often use to evaluate fluid response and further administration from 60 minutes until 24 hours of admission	Possible to choose from the same triggers as mentioned above
	Do you use other triggers for fluid administration from 60 minutes until 24 hours of admission?	Add others (text)
	How far ahead are you usually able to plan fluid administration to patients?	○ 30 min<br>○ 1-2 hours<br>○ 3-6 hours<br>○ 7-12 hours<br>○ 13-24 hours
**Clinical scenarios**<br><br>In the following section you will be given some clinical scenarios. You will be asked to decide on fluid administration for the fictive patient. We will ask you to answer as you would usually do in your daily clinical work.		
	Case 1: A previously, otherwise healthy 55-year-old woman (70 kg) was admitted with suspected pneumonia, with a history of cough and dyspnea through 14 days. She is slightly confused (GCS 14), BP 120/75, pulse rate 120, respiration rate 28, temperature 39.1°C and saturation 92% (3 liter/min nasal oxygen).<br>How much fluid would you administer within the first hour?	○ No fluids<br>○ 500 ml<br>○ 1000 ml<br>○ 1500 ml<br>○ 2000<br>○ 3000<br>○ 4000<br>○ Don't know
	Should fluid input and output be documented for this patient?	○ Yes<br>○ No<br>○ Don't know
	What did you base your decisions on fluid volumes on?	○ Knowledge and evidence in the field<br>○ Clinical judgement<br>○ Education<br>○ Experience<br>○ Patient history

		○ Other (please, elaborate)
	Case 2: A previously, otherwise healthy 55-year-old woman (70 kg) was admitted with suspected pneumonia, with a history of cough and dyspnea through 14 days. She is slightly confused (GCS 14), **BP 95/60**, pulse rate 120, respiration rate 28, temperature 39.1°C and saturation 92% (3 liter/min nasal oxygen).<br>How much fluid would you administer within the first hour?	○ No fluids<br>○ 500 ml<br>○ 1000 ml<br>○ 1500 ml<br>○ 2000<br>○ 3000<br>○ 4000<br>○ Don't know
	Should fluid input and output be documented for this patient?	○ Yes<br>○ No<br>○ Don't know
	Fluid administration should be evaluated again after …	○ 1 hour<br>○ 2-3 hours<br>○ 4-5 hours<br>○ 6-12 hours<br>○ 13-24 hours
	Case 1 and 2: I would chose the following fluid management strategy in the two cases, if electrolytes (Na, Ka, and Cl) were normal	○ NaCl (0.9% normal saline)<br>○ Ringers' (acetate or lactate)<br>○ Initially NaCl followed by Ringers'<br>○ NaCl and albumin<br>○ Ringers' and albumin<br>○ Kalium-natrium-glucose<br>○ Glucose<br>○ Don't know
	Case 3: A 55-year-old woman (70 kg) with known renal failure (habitual creatinine 200-220 µmol/l) was admitted with suspected pneumonia, with a history of cough and dyspnea through 14 days. She is slightly confused (GCS 14), BP 120/75, pulse rate 120, respiration rate 28, temperature 39.1°C and saturation 92% (3 liter/min nasal oxygen).	○ No fluids<br>○ 500 ml<br>○ 1000 ml<br>○ 1500 ml<br>○ 2000<br>○ 3000<br>○ 4000<br>○ Don't know

	How much fluid would you administer within the first hour?	
	Case 4: A 55-year-old woman (70 kg) with known heart failure (ejection fraction usually 30%) was admitted with suspected pneumonia, with a history of cough and dyspnea through 14 days. She is slightly confused (GCS 14), BP 120/75, pulse rate 120, respiration rate 28, temperature 39.1[illegible]C and saturation 92% (3 liter/min nasal oxygen).<br>How much fluid would you administer within the first hour?	○ No fluids<br>○ 500 ml<br>○ 1000 ml<br>○ 1500 ml<br>○ 2000<br>○ 3000<br>○ 4000<br>○ Don't know
Teaching and education<br>The following questions are focused on teaching and research in fluid administration of patients with suspected infections		
	When did you most recently attend an educational session on fluids?	○ 0-6 months<br>○ 7-12 months<br>○ 12 months<br>○ Never<br>○ Don't know
	I would like to learn more about fluid treatment of infected patients	○ Strongly agree<br>○ Agree<br>○ Neither or<br>○ Disagree<br>○ Strongly disagree
	There is a need for research and evidence within the fluid of fluid administration to ED patients with infections is	○ Strongly agree<br>○ Agree<br>○ Neither or<br>○ Disagree<br>○ Strongly disagree
**Use of vasopressors**<br>The following questions regard you opinion towards a potential implementation of vasopressor use in the emergency department administered through a peripheral intravenous catheter.		

This section was only available for physicians		
	Use of early vasopressors administered through a peripheral intravenous catheter in the ED should be implemented	○ Strongly agree<br>○ Agree<br>○ Neither or<br>○ Disagree<br>○ Strongly disagree
	I regard it possible to implement use of vasopressors administered through a peripheral intravenous catheter in the ED	○ Strongly agree<br>○ Agree<br>○ Neither or<br>○ Disagree<br>○ Strongly disagree
**Comments**		
	Please add comments or suggestions to improvements of fluid administration to patients with infections	Text

Table S1 Characteristics of the respondents		
	**Physicians (n=138)**	**Nurses (n=382)**
Hospital		
Regional Hospital Randers	31 (22%)	64 (17%)
Regional Hospital Viborg	26 (19%)	69 (18%)
Regional Hospital Herning	28 (20%)	77 (20%)
Regional Hospital Horsens	25 (18%)	41 (11%)
Aarhus University Hospital	28 (20%)	131 (45%)
For physicians only		
Primary specialties (currently in training in specialty or full training completed)		
Emergency medicine	99 (72%)	
Family medicine	24 (17%)	
Anesthesiology and intensive care	2 (1%)	
Dermatology	1 (1%)	
Cardiology	1 (1%)	
General surgery (abdominal)	1 (1%)	
Oncology	1 (1%)	
Pulmonary medicine	2 (1%)	
Nephrology	2 (1%)	
Neurosurgery	1 (1%)	
Orthopedic surgery	1 (1%)	
Rheumatology	1 (1%)	
Thoracic surgery	1 (1%)	
Ear-nose-throat surgery	1 (1%)	
For nurses only		
Further training besides nursing school[a]		
No		94 (25%)
Yes, foundation module of emergency medicine nursing specialization		141 (37%)
Yes, foundation and module 2 of emergency medicine nursing specialization		69 (18%)
Yes, alle three modules of emergency medicine nursing specialization		81 (21%)
Yes, specialized in anesthesia		3 (1%)
Yes, specialized in critical care		7 (2%)
Other		31 (8%)

All data are presented as n (%)
[a] it was possible to check more than one option

Table S2 Survey responses: Administration of fluids and use of guidelines		
	**Physicians (n=123)**	**Nurses (n=317)**
I would like to learn more about fluid treatment of patients with sepsis		
Strongly agree	92 (75%)	187 (59%)
Agree	30 (24%)	114 (36%)
Neither or	0	16 (5%)
Disagree	1 (1%)	0
Strongly disagree	0	0
There is a need for research and evidence within the field of fluid administration to ED patients with infections		
Strongly agree	45 (37%)	58 (18%)
Agree	38 (31%)	91 (29%)
Neither or	35 (29%)	155 (49%)
Disagree	4 (3%)	13 (4%)
Strongly disagree	1 (1%)	0
ED: emergency department,		

**Table S3 Survey responses from only physicians: Use of vasopressors**	
	**Physicians (n=123)**
Use of early vasopressors administered through a peripheral intravenous catheter in the ED should be implemented	
Strongly agree	13 (11%)
Agree	24 (20%)
Neither or	64 (52%)
Disagree	19 (15%)
Strongly disagree	3 (2%)
I regard it possible to implement use of vasopressors administered through a peripheral intravenous catheter in the ED	
Strongly agree	14 (11%)
Agree	24 (20%)
Neither or	56 (46%)
Disagree	22 (18%)
Strongly disagree	7 (6%)

ED: emergency department,

**Table S4 Triggers of fluid administration**						
	**To initiate fluids during the first 60 min of patient arrival**			**To evaluate fluid response and further administration from 60 minutes until 24 hours**		
	**All (n=520)**	**Nurses (n=382)**	**Physicians (n=138)**	**All (n=520)**	**Nurses (n=382)**	**Physicians (n=138)**
Blood pressure	469 (90%)	346 (91%)	123 (89%)	419 (81%)	306 (80%)	113 (82%)
Temperature	327 (63%)	277 (73%)	50 (36%)	217 (42%)	198 (52%)	19 (14%)
Patient history	203 (39%)	145 (38%)	58 (42%)	124 (24%)	83 (22%)	41 (30%)
Arterial or venous blood gas	182 (35%)	118 (31%)	64 (46%)	121 (23%)	70 (18%)	51 (37%)
Pulse rate	159 (31%)	105 (27%)	54 (39%)	107 (21%)	61 (16%)	46 (33%)
Fasting	156 (30%)	140 (37%)	16 (12%)	222 (43%)	199 (52%)	23 (17%)
Laboratory values[a]	133 (26%)	93 (24%)	40 (29%)	279 (54%)	201 (53%)	76 (57%)
Elasticity of the skin	129 (25%)	106 (28%)	23 (17%)	69 (13%)	56 (15%)	13 (9%)
Level of conciousness	107 (21%)	89 (23%)	18 (13%)	91 (18%)	74 (19%)	17 (12%)
qSOFA-score	104 (20%)	64 (17%)	40 (29%)	49 (9%)	33 (9%)	16 (12%)
Urine output and/or colour	94 (18%)	75 (20%)	19 (14%)	275 (53%)	193 (51%)	82 (59%)
Edema	82 (16%)	49 (13%)	33 (24%)	96 (18%)	66 (17%)	30 (22%)
Respiratory rate	80 (15%)	61 (16%)	19 (14%)	56 (11%)	40 (10%)	16 (12%)
SOFA-score	46 (9%)	29 (8%)	17 (12%)	35 (7%)	22 (6%)	13 (9%)
Capillary refill time	41 (8%)	24 (6%)	17 (12%)	18 (3%)	11 (3%)	7 (5%)
Mucus membranes	27 (5%)	14 (4%)	13 (9%)	36 (7%)	23 (6%)	13 (9%)
Stethoscopy pulmonis	25 (5%)	4 (1%)	21 (15%)	27 (5%)	7 (2%)	20 (15%)
Ultrasound: heart/lungs/VCI	17 (3%)	4 (1%)	13 (9%)	40 (8%)	18 (5%)	22 (16%)
Mean arterial pressure	14 (3%)	5 (1%)	9 (6%)	13 (3%)	5 (1%)	8 (6%)
Mottling	8 (2%)	3 (1%)	5 (4%)	4 (1%)	1 (0%)	3 (2%)
Passive leg raise	7 (1%)	1 (0%)	6 (4%)	5 (1%)	1 (0%)	4 (3%)
Temperature of the extremities	7 (1%)	5 (1%)	2 (1%)	6 (1%)	5 (1%)	1 (1%)
Jugular vein distension	5 (1%)	4 (1%)	1 (1%)	10 (2%)	8 (2%)	2 (1%)

All results are in number (%). Each respondent chose the 5 most frequently used triggers of fluid administration in daily practice; therefore percentages cannot be summed. The triggers are sorted by response frequency of the overall column for the initial trigger. ED: emergency department, VCI: vena cava inferior, qSOFA-score: quickSOFA, SOFA: Sequential (Sepsis related) Organ Failure Assessment

[a] laboratory values included creatinine, albumin etc.

Figure S1

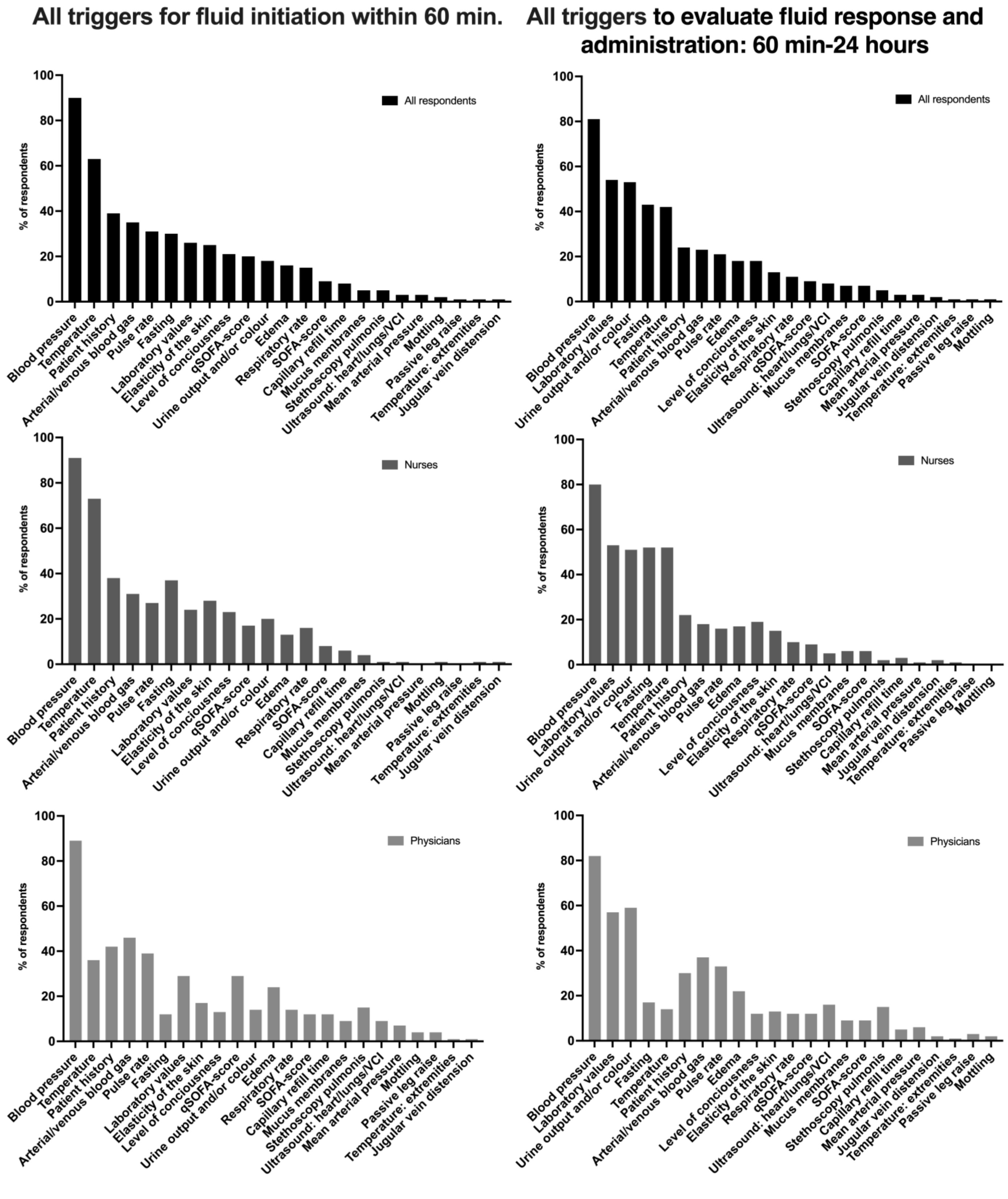

Figure S1 All figures to the left show the distribution of used triggers of fluid initiation within 60 minutes of patient arrival grouped as all respondents, only nurses and only physicians. To the right, triggers to evaluate fluid response and further administration from 60 minutes until 24 hours

from patient arrival are shown. The Y-axis shows percentages of respondents who chose the trigger. Each respondent had to choose the five most often used triggers in daily practice. The triggers are ordered by frequency in all respondents for both time intervals.
